# The impact of primary tumor location on efficacy of cetuximab in metastatic colorectal cancer patients with different Kras status: a systematic review and meta-analysis

**DOI:** 10.18632/oncotarget.19022

**Published:** 2017-07-05

**Authors:** De-Dong Cao, Hui-Lin Xu, Xi-Ming Xu, Wei Ge

**Affiliations:** ^1^ Department of Oncology, RenMin Hospital of Wuhan University, Wuhan, China; ^2^ Department of Oncology, The Fifth Hospital of Wuhan, Wuhan, China

**Keywords:** primary tumor location, cetuximab, colorectal cancer, Kras, meta-analysis

## Abstract

**Objective:**

To assess the prognostic role of primary tumor location along with Kras status in metastatic colorectal cancer (mCRCs) treated with cetuximab.

**Materials and Methods:**

Databases of EMBASE, Pubmed, the Cochrane library, China National Knowledge Infrastructure and other databases from inception to July 2016 were searched. Randomized controlled trial (RCT) and/or retrospective studies of influence of primary tumor location on efficacy of cetuximab in patients with mCRC were identified. The primary endpoint was progression-free survival (PFS), and the secondary endpoints were overall survival (OS), overall response rate (ORR) and disease control rate (DCR).

**Results:**

Ten studies including 2977 cases were finally included. The results of meta-analysis were in favor of cetuximab to patients with left-sided colorectal cancer in terms of OS (HR = 0.52, 95% CI: 0.40–0.66; *p* < 0.01), PFS (HR = 0.64, 95% CI: 0.58–0.70; *p* < 0.01), and ORR (OR = 2.17, 95% CI: 1.57–2.99; *p* < 0.01). Patients with right-sided CRC gained less benefit from cetuximab in terms of OS (HR = 1.89, 95% CI: 1.43–2.50; *p* < 0.01), compared with left-sided CRC. Regarding Kras status, left-sided mCRC with wild type Kras had better PFS (HR = 0.61, 95% CI: 0.51–0.74; *p* < 0.01) and OS (HR = 0.49, 95% CI: 0.35–0.69; *p* < 0.01) than right-sided cases when treated with cetuximab. We also found that cetuximab was both significantly effective in different treatment lines and regions when comparing by primary tumor locations (*p* < 0.01).

**Conclusions:**

mCRC Patients with left-sided, wild type Kras have a better prognosis than those with right-sided diseases when treated with cetuximab. The clinical application of cetuximab should be determined by the primary tumor location and molecular gene mutation status.

## INTRODUCTION

Colorectal cancer (CRC) is one of the most common malignances worldwide, accounted for 9.7% of all incident cancers in 2015 [1]. In recent years, a decline in CRC mortality have been observed in North America, European countries, and Japan [1, 2]. These improvements in reducing CRC caused death are thought to be achieved by adequate prevention, early diagnosis, and effective treatment regimens [3]. With regards to metastatic CRC, the application of molecular targeted therapies, such as cetuximab, is reported to improve clinical outcome in this population [4, 5]. However, the efficacy of cetuximab is determined by several clinical and molecular characteristics and not all of these metastatic CRC patients are benefited from this agent, indicating that application of cetuximab should be considered according to personalized information [6, 7]. Therefore, it is necessary to find specific clinical feature and biomarker to identify patients who can gain favorable outcome from cetuximab administration.

Meta-analysis is a perfect method to help identify existent data across individual studies. Previously, several meta-analysis have tried to determine possible factors affecting the outcome of metastatic CRC, and their findings are encouraging [8–14]. Although these meta-analysis focus on primary tumor location or gene mutation status in metastatic CRC patients, none has focused specifically on the impact of primary tumor location in prognosis of metastatic CRC patients treated with cetuximab. Thus it is time to explore the clinical importance of primary tumor site in affecting the efficacy of cetuximab in this population.

In 2015, Lee G.H. et al. [15] published a systematic review evaluating the importance of tumor location in management of CRC. They defined proximal or right-sided colon cancer (RCC) as cancers of the caecum, ascending and transverse colon up to the splenic flexure, and distal or left-sided CRC (LCRC) as cancers of the descending and sigmoid colon and rectum [15]. They concluded that primary tumor subsite associated various factors caused the differences in presentation and clinical outcome in LCRC and RCC. They suggested that management of CRC should be determined by biological and molecular characteristics based on the anatomical site. One year later, Masashi Yahagi et al. [9] conducted a meta-analysis to quantify the prognostic differences between left-sided colon cancer (LCC) and right-sided colon cancer (RCC). In their study, the LCC was defined as tumors located from the splenic flexure to the sigmoid colon or recro-sigmoid colon, and RCC referred to cancers in the cecum, ascending colon or transverse colon. A total of fifteen trials comparing the prognosis of colon cancer according to tumor location with or without adjuvant chemotherapy were included and assessed. The combined data showed that RCC had a worse outcome than LCC in terms of OS (hazard ratio = 1.14, *p* < 0.01). It is concluded that tumor location is associated with prognosis in colon cancer patients, and LCC population has a better prognosis than RCC with regards to OS. The authors suggested that RCC should have a distinctive treatment strategy and standardized management for different colon cancer location is needed. This study neither provide information about the impact of tumor location on efficacy of treatments such as cetuximab, nor the molecular data (Kras for example) in different tumor location.

Kars is one of the best known biomarker influencing the effectiveness of cetuximab [6, 8, 10]. Few months ago, Li Lin et al. [8] used meta-analysis to investigate the cetuximab effectiveness in patients with different Kras status when combined with chemotherapy. Nine studies were eligible for the final analysis. The results determined that addition of cetuximab significantly improved OS in Kras exon 2 wild-type patients (HR = 0.87, *p* = 0.004). No significant differences in terms of OS and PFS were observed in tumors with Kras exon 2 mutation. The study concluded that cetuximab combined with chemotherapy led to a longer OS in metastatic CRC patients who lacked Kras mutation. They suggested cetuximab should be used in metastatic CRC patients with wild-type Kras status. However, this study only assessed the efficacy of cetuximab based on Kras status, but not on primary tumor location.

Taken together, published meta-analyses suggest that colon cancer patients with different tumor location have different prognosis, and patients with wild-type Kras have a better outcome when using cetuximab. Although the personalized treatment for cancer is an active area of investigation, these existing meta-analysis are not capable of providing encouraging evidence for the use of cetuximab based on both primary tumor location and Kras status. In addition, analysis about the efficacy of cetuximab according to treatment line is also lack of combined evidence.

With regards to the concerns mentioned above, we performed this meta-analysis to examine clinical studies regarding the impact of primary tumor location on the efficacy of cetuximab in metastatic CRC patients with different Kras status. With this purpose, we expected to identify all relevant studies of metastatic CRC patients who were treated with cetuximab and had survival analysis based on both cancer location and gene status, and analyzed these data by meta-analysis technology.

## MATERIALS AND METHODS

### Search strategy

This meta-analysis was performed with the Preferred Reporting Items for Systematic Reviews and Meta-Analyses guidelines (PRISMA) [16]. This study did not have registration information. Electronic databases including EMABSE, the cochrane library, Pubmed, Wanfang database, China National Knowledge Infrastructure, and American Society of Clinical Oncology meeting abstract were chosen and searched for clinical studies comparing prognosis of metastatic CRC patients with LCRC vs. RCC who were treated with cetuximab, published between inception and September 2016. The search terms of [“colonic neoplasm” or “carcinoma, colorectal” or “tumor, colorectal”], [“prognosis”, or “survival analysis”], [“cetuximab” or “anti-EGFR agents”], [“KRAS” or “K-ras”] and [“tumor location” or “tumor site” or “left” or “right” or “subsite”] were used with different combinations in these databases. The “similar articles” function in Pubmed was used. No language limitation was applied in this study. We also included conference abstracts if sufficient data provided.

### Study selection

The primary endpoint of this meta-analysis was progression-free survival (PFS), and the secondary endpoints were overall survival (OS), overall response rate (ORR) and disease control rate (DCR) based on Kras status or not. All of the included studies had a clear and adequate definition of PFS, OS, ORR and DCR. Retrospective or prospective cohort studies or randomized controlled trials (RCT) that determining the association between primary tumor location and efficacy of cetuximab were included. The inclusion criteria of this meta-analysis were: (1) clinical studies performed in metastatic CRC patients, either mentioned in form of full-text or abstract; (2) metastatic CRC patients treated with cetuximab in combination with traditional chemotherapy; (3) adequate definition of RCC and LCRC; (4) trials comparing prognosis in patients with LCRC vs. RCC; (5) studies reported hazard ratio (HR) and 95% confidence interval (CI) for PFS and/or OS according to primary tumor location, or HR could be calculated using the data provided; (6) secondary endpoints were overall response rate (ORR) or disease control rate (DCR). Studies including reviews, comments, letters, case reports and meta-analysis were excluded.

### Data extraction and quality assessment

Original data in each eligible trial was adequately extracted and identified by two reviewers (Dedong Cao and Huilin Xu) independently. Disagreements in the process of data extraction were resolved by consensus. The baseline characteristics and clinical outcomes including number of participants, publication data, age, gender, country, performance status, method of randomization if RCT, treatment line of cetuximab, chemotherapy regimen, Kras status, PFS, OS, ORR and DCR were extracted from included studies. If HR and its 95% CI were not directly provided by the original study, the method provided by Parmar et al [17] was introduced to calculate these data. The first author’ name and year of publication were applied to identify each study during the process of analysis.

For quality assessment of included studies, the Newcastle Ottawa Scale (NOS) [18] recommended by the Cochrane Non-Randomized Studies Methods Working Group was used. Based on the NOS, studies were assessed based on three broad perspectives: selection (four criteria), comparability (one criteria), and outcome (three criteria). Given the variability in quality among observational studies, a high-quality study was defined if it had six or more NOS criteria stars, with a possible range of 1 to 9. A high quality study was defined as low-risk of bias consisting of selection, performance, and reporting. This part of work was performed by two reviewers (Dedong Cao and Huilin Xu), independently. If discrepancies in quality assessment of included studies emerged, a third reviewer (Wei Ge) was involved to resolve it.

### Statistical analysis

The RevMan 5.3 software provided by the Cochrane Collaboration was used to conduct all of the statistical analysis, following the introduction of the Cochrane Collaboration for meta-analysis. The endpoints in the pooled analysis were PFS and OS in the setting of different tumor location, and they were showed by HRs with 95% CI. These comparisons were also performed in sub-group analysis in terms of Kras status and line of treatment. The logarithms of HRs (logHRs) were calculated. If the HRs and their corresponding 95% CI were provided, logHRs and the standard error were used in the combined analysis. Sensitivity analysis aiming to examine the heterogeneity across the included article was also performed. Prior to the pooled synthesis, I^2^ statistics were calculated to judge the degree of heterogeneity. Heterogeneity was defined according to the Cochrane Handbook [19]: I^2^% = 0% to 40%, might be low risk of heterogeneity; 30% to 60%: may represent moderate heterogeneity; 50% to 90%: may represent substantial heterogeneity; 75% to 100%: considerable heterogeneity. If the heterogeneity was low, a fixed-effects meta-analysis was performed, otherwise the random-effects model was applied. HRs and associated 95% CIs were used to quantify the influence of tumor location on endpoints of PFS and OS. A combined HR < 1 indicated a better survival for the LCRC patients. Pooled estimates of OR were calculated for RCC vs. LCRC in terms of ORR and DCR using Mantel-Haenszel OR method [19]. The funnel plots were used to assess the degree of publication bias. All statistical tests were two-sided, and it was regarded as statistical significant if the *p* < 0.05, except for heterogeneity analysis.

## RESULTS

### Study selection and characteristics

**Figure 1:**
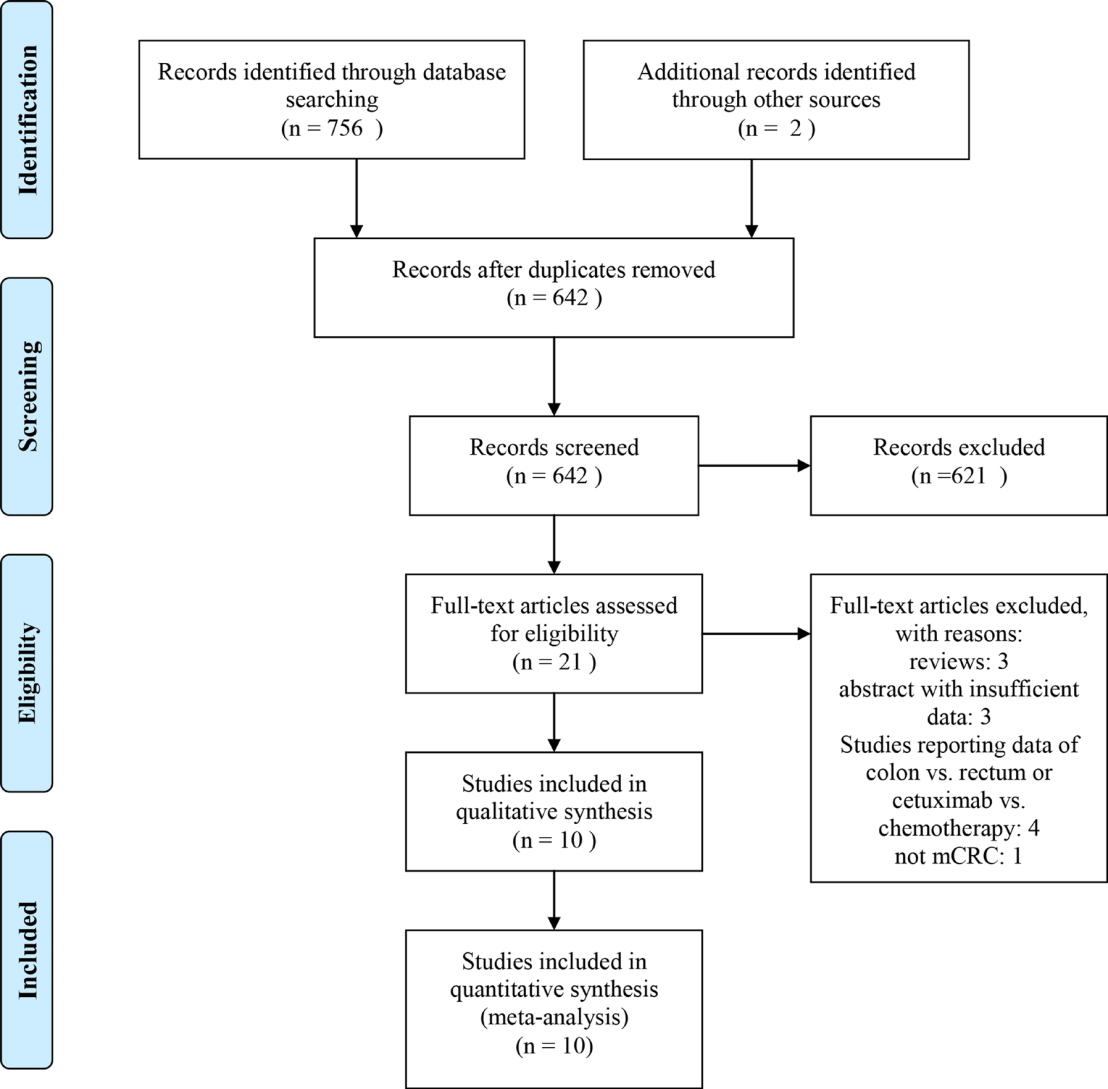
Flow diagram of searching for eligible studies

A total of 758 studies were retrieved initially after systematically searching the selected databases. Of these articles, 116 were duplicates, resulting in 642 abstracts. After reviewing, a number of 21 clinical studies were further evaluated. Among them, 11 articles were excluded as they failed to meet the inclusion criteria. Finally 10 clinical trials [20–29] were included in the final meta-analysis (Figure 1). Of included studies, all of them [20–29] were retrospective studies compared the impact of tumor location on the outcome of cetuximab in mCRC patients. 4 of them were ASCO meeting abstracts compared patients who received cetuximab could gain a better benefit according to their primary tumor sites [26–29]. One study was RCT compared the disease-free survival (DFS) of stage III colon cancer patients who treated with standard adjuvant oxaliplatin, fluorouracil, and leucovorin chemotherapy (FOLFOX4) with or without cetuximab [30]. However, this study was excluded as the participants were stage III, and received surgery. Specially, additional data was provided by the MERCK company. There were five studies [20, 21, 23, 24, 28] from Eastern countries, and five [22, 25–27, 29] from Western countries. Eight studies [20, 22, 24–29] reported data of HR directly, while data of HR was calculated indirectly from survival curves from two studies [21, 23]. Additional updated data of the FIRE 3 and the CRYSTAL trials were obtained from one retrospective analyses study [31]. The baseline characteristics of included studies are presented in Table 1. The quality of included studies is summarized in Table 2. Additional information are presented in Supplementary Table 1.

**Table 1:** Baseline characteristics of included studies

Study	Year	Country	Primary tumor site	Treatment regimen	No. of patients	Median age (range)	Male/ Female	performance status	Line of treatment	Outcome
Wang Jue	2016	China	left-sided	Cetuximab +FOLFOX/FOLFIRI	32	59 (38–76)	22/10	NA	First-line	ORR, PFS
			right-sided	Cetuximab +FOLFOX/FOLFIRI	16	56 (34–74)	11/5	NA	First-line	ORR, PFS
Feng Wang	2015	China	left-sided	Cetuximab +mFOLFOX-6/XELOX/ FOLFIRI	145	NA	98/47	ECOG (0/1/2): 78/61/6	First-line/second-line	ORR, OS, PFS
			right-sided	Cetuximab +mFOLFOX-6/XELOX/ FOLFIRI	61	NA	40/21	ECOG (0/1/2): 24/32/5	First-line/second-line	ORR, OS, PFS
von Einem JC	2014	Germany	left-sided	Cetuximab + CaPIRI/ CaPOX	100	63 (32–77)	77/23	Karnofsky > 90/70 + 80/NR): 73/25/2	First-line/second-line	ORR, OS, PFS
			right-sided	Cetuximab + CaPIRI/ CaPOX	46	61 (47–74)	28/18	Karnofsky (> 90/70 + 80/NR): 32/14/0	First-line/second-line	ORR, OS, PFS
Kuo-Hsing Chen	2016	China	left-sided	Cetuximab + chemotherapy	765	60 (22–96)	591/378	NR	First-line	OS, TTD
			right-sided	Cetuximab + chemotherapy	136	60 (22–96)		NR	First-line	OS, TTD
Rui Qin	2014	China	left-sided	Cetuximab +FOLFOX/FOLFIRI/XELOX	63	56 (21–86)	64/26	NR	First-line/second-line	ORR, OS, PFS
			right-sided	Cetuximab +FOLFOX/FOLFIRI/XELOX	27	56 (21–86)		NR	First-line/second-line	ORR, OS, PFS
Moretto R	2016	Italy	left-sided	anti-EGFR or cetuximab-irinotecan	61	NA	NA	NA	First-line/second-line	ORR, PFS
			right-sided	anti-EGFR or cetuximab-irinotecan	14	NA	NA	NA	First-line/second-line	ORR, PFS
Alan P. Venook	2016	USA	left-sided	Cetuximab +FOLFOX/FOLFIRI	689	59	NA	NA	First-line	OS, PFS
			right-sided	Cetuximab +FOLFOX/FOLFIRI	342	59	NA	NA	First-line	OS, PFS
Eric Van Cutsem	2016	Germany	left-sided	Cetuximab + FOLFIRI	142	60	NA	NA	First-line	ORR
			right-sided	Cetuximab + FOLFIRI	33	60	NA	NA	First-line	ORR
Heinemann V	2014	multi-center	left-sided	Cetuximab + FOLFIRI	157	NA	NA	NA	Second-line	PFS, OS
			right-sided	Cetuximab + FOLFIRI	38	NA	NA	NA	Second-line	PFS, OS
Yu Sunakawa	2016	Japan	left-sided	Cetuximab + FOLFOX/SOX	90	NA	NA	NA	First-line	ORR, OS, PFS
			right-sided	Cetuximab + FOLFOX/SOX	20	NA	NA	NA	First-line	ORR, OS, PFS

Abbreviations: TTD, time to treatment discontinuation; OS, overall survival; ECOG, Eastern Cooperative Oncology Group; RCC, right-sided colon cancer; LCRC, left-sided colorectal cancer; BSC, Best supportive care; ORR, overall response rate; DFS, disease free survival; PFS, progression free survival; NA, not available; NR, not reported; WHO, world health organization; PS, performance status; EGFR, epidermal growth factor receptor.

**Table 2:** Methodological quality assessment of included studies by NOS

Study	Year	Selection	Comparability	Outcome
Wang Jue	2016	***	*	**
Feng Wang	2015	***	**	***
von Einem JC	2014	**	*	**
Kuo-Hsing Chen	2016	***	**	***
Rui Qin	2014	***	*	***
Moretto R	2016	****	*	***
Venook AP	2016	****	**	***
Heinemann V	2014	***	**	***
Van Cutsem E	2016	***	**	***
Yu Sunakawa	2016	***	**	**

Note: A study can be awarded a maximum of one star for each numbered item within the Selection and Outcome categories. A maximum of two stars can be given for Comparability, according to the instruction of NOS.

### Results of meta-analysis

#### PFS between LCRC vs. RCC in mCRC patients treated with cetuximab

**Figure 2:**
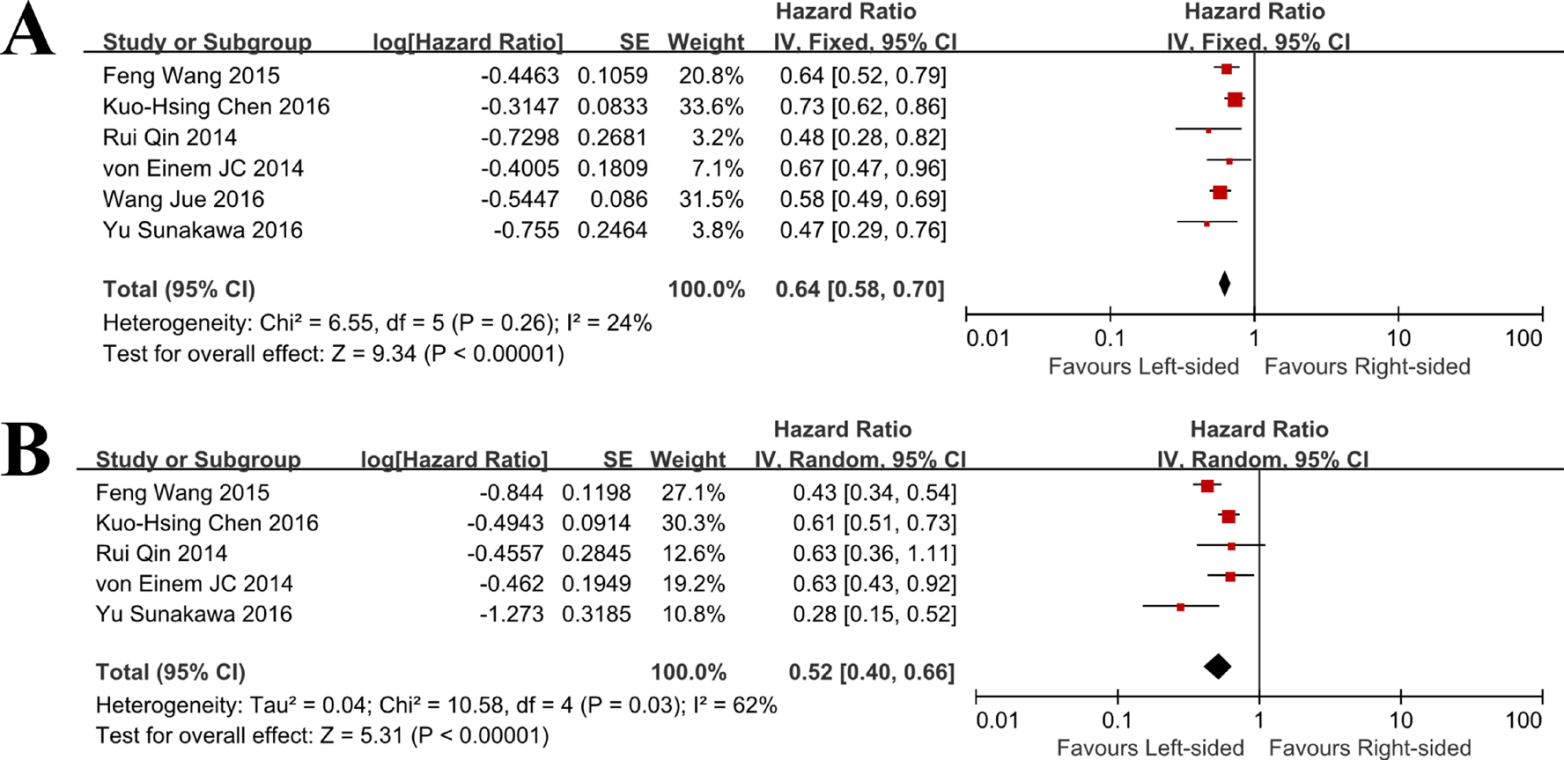
Progression free survival and overall survival outcomes for cetuximab by primary tumor location in patients with metastatic colorectal cancer (A) Forest plot of the Hazard ratio for pooled PFS in patients with LCRC vs. RCC (**B**) Forest plot of the Hazard ratio for pooled OS in patients with LCRC vs. RCC. LCRC, left-sided colorectal cancer; RCC, right-sided colorectal cancer).

A combined analysis of PFS between LCRC and RCC in mCRC patients was performed. A total of 6 studies reporting HRs for PFS were involved. As there was no significant evidence of heterogeneity within these studies (I^2^% = 24%, *p* = 0.26), the fixed effect model was used for this analysis. The aggregated results showed that there was significant PFS benefit from cetuximab in patients with LCRC when compared with RCC patients who also received cetuximab (HR = 0.64, 95% CI = 0.58–0.70; *p* < 0.001; Figure 2).

There were three trials provided data of PFS when comparing RCC versus LCRC in mCRC patients treated with cetuximab. As indicated by the I^2^% = 75%, we considered there was significant heterogeneity across these studies, so the random effect model was applied. As shown by the results, patients with RCC had a combined HR of 2.11 (95% CI = 1.35–3.30; *p* = 0.001) than those with LCRC, suggesting RCC patients had a significantly higher risk of progressive disease.

Subgroup analyses based on different Kras status were performed. Cetuximab significantly improved PFS for LCRC patients with wild type Kras (HR = 0.61, 95% CI = 0.51–0.74; *p* < 0.001), but not mutated Kras (HR = 1.01, 95% CI = 0.56–1.82; *p* = 0.97), when compared to RCC patients (Table 3). Subgroup analyses based on treatment line of cetuximab were performed. The pooled results indicated that LCRC could significantly gain benefit from cetuximab regardless of line of treatment, when compared with RCC patients (*P* < 0.01, Table 3).

**Table 3:** Summary of subgroup analyses based on different clinical or molecular features in patients with different primary tumor sites

Event		HR	95% CI	*P* value
All patients				
PFS				
	LCRC vs. RCC	0.64	0.58–0.70	< 0.001
	RCC vs. LCRC	2.11	1.35–3.30	0.001
OS				
	LCRC vs. RCC	0.52	0.40–0.66	< 0.001
	RCC vs. LCRC	1.89	1.43–2.50	< 0.001
Wild type Kras				
PFS				
	LCRC vs. RCC	0.61	0.51–0.74	< 0.001
	RCC vs. LCRC	1.29	0.69–2.4	0.42
OS				
	LCRC vs. RCC	0.49	0.35–0.69	< 0.001
	RCC vs. LCRC	1.89	1.43–2.50	< 0.001
Mutated Kras				
PFS				
	LCRC vs. RCC	1.01	0.56–1.82	0.97
	RCC vs. LCRC	-	-	-
OS				
	LCRC vs. RCC	1.30	0.68–2.49	0.43
	RCC vs. LCRC	1.88	0.86–4.10	0.11
Regions				
PFS				
Western	LCRC vs. RCC	0.67	0.47–0.96	0.03
Eastern	LCRC vs. RCC	0.63	0.58–0.70	< 0.001
OS				
Western	LCRC vs. RCC	0.63	0.3–0.92	< 0.001
Eastern	LCRC vs. RCC	0.53	0.46–0.60	< 0.001
Line of cetuximab				
PFS				
First-line	LCRC vs. RCC	0.65	0.54–0.77	< 0.001
Other lines	LCRC vs. RCC	0.48	0.28–0.82	< 0.001
OS				
First-line	LCRC vs. RCC	0.50	0.35–0.73	< 0.001
Other lines	LCRC vs. RCC	-	-	-

Abbreviations: RCC, right-sided colon cancer; LCRC, left-sided colorectal cancer; OS, overall survival; ORR, overall response rate; PFS, progression free survival; HR, hazard ratio; mu, mutated; wt, wild type; CT, chemotherapy.

### OS between LCRC vs. RCC in mCRC patients treated with cetuximab

Five studies reported data on OS. The random effect model was applied as indicated by the I^2^% = 62% (*p* = 0.03). The pooled results suggested that use of cetuximab could significantly prolong OS in patients with LCRC when compared to RCC patients (HR = 0.52, 95% CI = 0.40–0.66; *P* < 0.001; Figure 2). There were four trials provided data of OS when comparing RCC versus LCRC in mCRC patients treated with cetuximab. A value of 67% was observed for I2%, so the random effect model was used. As shown by the results, patients with RCC had a combined HR of 1.89 (95% CI = 1.43–2.50; *p* < 0.001) than those with LCRC, suggesting RCC patients had a risk of 1.89 times for progressive disease.

In subgroup analyses, a significant improvement in OS was found for LCRC patients with wild type Kras (HR = 0.49, 95% CI = 0.35–0.69; *P* < 0.001), but not for patients with mutated Kras (HR = 1.30, 95% CI = 0.68–2.49; *P* = 0.43), when compared to RCC with different Kras status. Similarly, LCRC had a significantly prolonged OS after cetuximab treatment regardless of treatment line, when compared with RCC patients (*P* < 0.001, Table 3).

### ORR and DCR between LCRC vs. RCC in mCRC patients treat with cetuximab

**Figure 3:**
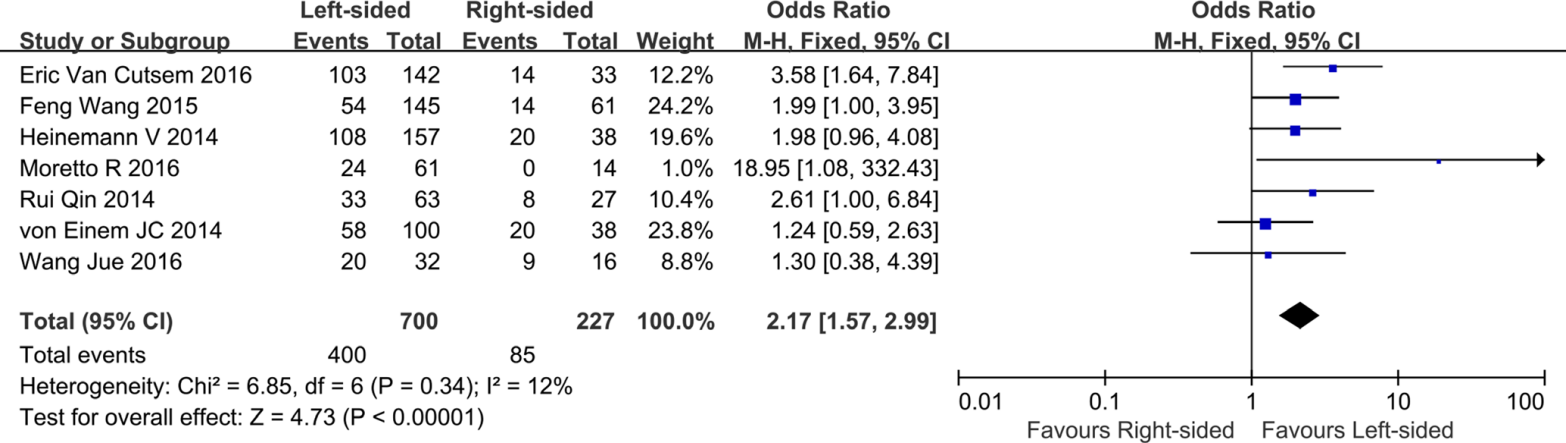
Overall response outcomes for cetuximab by primary tumor location in patients with metastatic colorectal cancer (forest plot of the hazard ratio for pooled overall response in patients with LCRC vs. RCC; LCRC, left-sided colorectal cancer; RCC, right-sided colorectal cancer)

Seven trials reported data on ORR (I^2^% = 1^2^%, *p* = 0.34). The combined outcomes indicated that metastatic LCRC patients treated with cetuximab had a better ORR when compared to those with RCC (OR = 2.17, 95% CI = 1.57–2.99; *P* < 0.001; Figure 3). Based on data of DCR reported by four studies, the synthesized DCR suggested that there was a high risk of heterogeneity across selected studies, even after sensitivity analysis. As a result, the combined analysis of DCR was abandoned. We did not perform subgroup analyses of ORR or DCR based on the primary tumor location due to insufficient data.

### Publication bias

**Figure 4:**
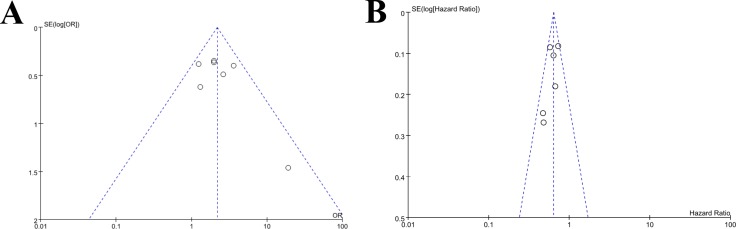
Funnel plots of publication bias in meta-analysis of overall response and progression free survival (**A**) Funnel plot for overall response; (**B**) Funnel plot for progression free survival).

The Egger's test and funnel plot were performed to assess the publication bias. As illustrated by Figure 4, there were no significant publication bias (*p* > 0.05) across the included studies as showed by the Egger's test with regards to the combined analysis of ORR and PFS.

## DISCUSSION

The current meta-analysis synthesized outcome data from 10 clinical studies elevating the prognosis of LCRC vs. CRR in metastatic patients who received cetuximab regimen. The combined results showed that LCRC patients had a better prognosis in terms of PFS, OS, ORR and DCR than patients with RCC, and this advantage was more obviously in LCRC patients with wild type Kras. The addition of cetuximab significantly improved prognosis of LCRC patients when combined with chemotherapy than chemotherapy alone. In contrast, in RCC patients with Kras mutation, no statistically significant improvement in OS but not PFS was found. These findings demonstrate that primary tumor site is evidently associated with clinical outcomes in mCRC patients. To our knowledge, this is the first meta-analysis based evidence to evaluate the role of primary tumor location on the prognosis of RCC patients treated with cetuximab.

The present study demonstrates that adding cetuximab to LCRC patients could result in clinical improvements in PFS, OS and ORR, significantly. For patients containing wild type Kras, longer PFS and OS were found in LCRC. Adding cetuximab therapy to traditional chemotherapy or best supportive care could also benefit patients with LCRC with regards to PFS. No OS benefit from combined treatments was observed in patients with RCC and mutated Kras status. Compared with clinical outcomes of LCRC and wild type Kras in patients treated with cetuximab, RCC and mutation of Kras are predictors of less sensitivity in terms of PFS, OS, and ORR. The sub-group analysis confirmed that LCRC patients could also benefit from cetuximab regardless of treatment line of cetuximab.

The findings of the present meta-analysis are in accordance with those of meta-analyses published by other reviewers [9, 32]. In the study conducted by Yahagi, M. et al. [9], they compared the prognostic differences between RCC and LCC, and the results showed that patients with RCC had a worse prognosis than those with LCC in OS. Their subgroup analyses showed that significant prognostic differences existed in Western countries vs. Eastern countries, nationwide database, and a stage-adjusted analysis. They concluded that tumor location was related to prognosis in CRC patients, and RCC had a worse prognosis than those with LCC with regard to OS. Another meta-analysis performed by Nitsche U et al. [32], they included 1319 patients who underwent surgery for colon cancer, and investigated the differences between tumors of the right and left colon. They defined tumors between the ileocecal valve and the hepatic flexure as RCC, and tumors between the splenic flexure and the rectum as LCC. The results demonstrated that RCC had a higher cause specific mortality risk and DFS. RCCs were more often showed Kras mutation and microsatellite instable than those with LCCs. They concluded that RCC and LCC differed significantly regarding clinical and molecular features and other factors. Our results also demonstrated that RCC patients had poor efficacy to cetuximab when compared with LCRC, although these meta-analyses did not include patients treated with cetuximab in combination with chemotherapy.

Several meta-analyses [8, 14, 33] confirm that Kras status is a good predictor of response to cetuximab therapy. These studies indicated that cetuximab should be used for mCRC with wild type Kras. In our study, we observed that participants with wild type Kras could benefit more from use of cetuximab than those with mutant Kras, especially in LCRC patients. Meanwhile, no significant OS benefit was found from cetuximab in the treatment of RCC with Kras mutation status. However, this benefit was mostly observed upon the mutation status of exon 2 in Kras. For patients with certain specific mutations in Kras, a greater sensitivity to anti-EGFR agents was found, when compared with other Kras mutations [10].

There are several clinical and research indications within the present meta-analysis. Clinically, the results suggest that the primary tumor location and gene mutation status should be examined prior to the initiation of cetuximab containing regimen. For mCRC patients, testing of Kras mutation status is suggested to be undertaken in order to gain valuable predictive information that may help making treatment decisions. RCC patients are suggested to receive personalized molecular targeted therapy distinctively from that of LCRC, as they benefited less from the cetuximab treatment. The recommended management for mCRC by primary tumor location is needed. Recently, a wide variety of tumor sample based studies [34, 35] aiming to illustrate the differences responsible for the different outcomes between LCRC and RCC has been published, however, the underlying mechanisms for the different responses of different tumor location to cetuximab in combination with chemotherapy are not fully illuminated. Therefore, researches are encouraged to understand the gene or molecular distinctions between individual patients, and this will help mCRC patients achieve the best efficacy after targeted molecular therapies.

However, there were several limitations in this meta-analysis. First, various variations existed within the included studies, including study design, baseline characteristics of participants, treatment regimen, line of treatment, follow-up intervals, analysis method and reporting types. All of these factors could influence the pooled results of this meta-analysis. Second, not of all the included studies directly reported the HRs and associated 95% CIs. Some of the HRs and 95% CIs had to be calculated based on the survival curves presented in the full-text articles, indirectly. Therefore, these less reliable data potentially affected the findings of the present study. Moreover, HRs for OS were only reported in a few studies, further limited the reliance of our findings. Third, three presentations from ASCO meetings were included. The results of the abstracts may differ from the full publication due to update of outcomes. Thus, we used random effect model to attenuate these limitations when it was necessary.

In conclusion, this meta-analysis indicates that left-sided colorectal cancer patients with wild type Kras have a better prognosis of survival when treated with cetuximab. This meta-analysis also reveals that RCC is associated with poorer response and survival in Kras mutation patients treated with cetuximab. The primary tumor location and Kras status should be determined prior to the initiation of administration of cetuximab in patients with mCRC. As the limited quality and number of clinical trials included in this meta-analysis, the findings should be further determined by more randomized controlled studies.

## SUPPLEMENTARY TABLE



## References

[1] Aran V, Victorino AP, Thuler LC, Ferreira CG (2016). Colorectal Cancer: Epidemiology, Disease Mechanisms and Interventions to Reduce Onset and Mortality. Clin Colorectal Cancer.

[2] Favoriti P, Carbone G, Greco M, Pirozzi F, Pirozzi RE, Corcione F (2016). Worldwide burden of colorectal cancer: a review. Updates Surg.

[3] Arnold M, Sierra MS, Laversanne M, Soerjomataram I, Jemal A, Bray F (2016). Global patterns and trends in colorectal cancer incidence and mortality. Gut.

[4] Modest DP, Stintzing S, von Weikersthal LF, Decker T, Kiani A, Vehling-Kaiser U, Al-Batran SE, Heintges T, Lerchenmuller C, Kahl C, Seipelt G, Kullmann F, Stauch M (2015). Impact of Subsequent Therapies on Outcome of the FIRE-3/AIO KRK0306 Trial: First-Line Therapy With FOLFIRI Plus Cetuximab or Bevacizumab in Patients With KRAS Wild-Type Tumors in Metastatic Colorectal Cancer. J Clin Oncol.

[5] Primrose J, Falk S, Finch-Jones M, Valle J, O’Reilly D, Siriwardena A, Hornbuckle J, Peterson M, Rees M, Iveson T, Hickish T, Butler R, Stanton L (2014). Systemic chemotherapy with or without cetuximab in patients with resectable colorectal liver metastasis: the New EPOC randomised controlled trial. Lancet Oncol.

[6] Shen H, Yang J, Huang Q, Jiang MJ, Tan YN, Fu JF, Zhu LZ, Fang XF, Yuan Y (2015). Different treatment strategies and molecular features between right-sided and left-sided colon cancers. World J Gastroenterol.

[7] Sinicrope FA, Okamoto K, Kasi PM, Kawakami H (2016). Molecular Biomarkers in the Personalized Treatment of Colorectal Cancer. Clin Gastroenterol Hepatol.

[8] Lin LI, Chen LL, Wang Y, Meng XY, Liang C, Zhou FX (2016). Efficacy of cetuximab-based chemotherapy in metastatic colorectal cancer according to RAS and BRAF mutation subgroups: A meta-analysis. Mol Clin Oncol.

[9] Yahagi M, Okabayashi K, Hasegawa H, Tsuruta M, Kitagawa Y (2016). The Worse Prognosis of Right-Sided Compared with Left-Sided Colon Cancers: a Systematic Review and Meta-analysis. J Gastrointest Surg.

[10] Li XX, Liang L, Huang LY, Cai SJ (2015). Standard chemotherapy with cetuximab for treatment of colorectal cancer. World J Gastroenterol.

[11] Lu X, Chen X, Sun J, Gao P, Song Y, Huang X, Luo Y, Chen P, Wang Z (2015). Polymorphism in epidermal growth factor is related to clinical outcomes of metastatic colorectal cancer patients treated with cetuximab: a systematic review and meta-analysis. Int J Clin Exp Med.

[12] Zhu YL, Lou J, Guo JY, Huang Z, Lv SW (2014). A meta analysis of cetuximab plus oxaliplatin based chemotherapy regimen for metastatic colorectal cancer. Indian J Cancer.

[13] Zhou SW, Huang YY, Wei Y, Jiang ZM, Zhang YD, Yang Q, Xie DR (2012). No survival benefit from adding cetuximab or panitumumab to oxaliplatin-based chemotherapy in the first-line treatment of metastatic colorectal cancer in KRAS wild type patients: a meta-analysis. PLoS One.

[14] Tsoukalas N, Tzovaras AA, Tolia M, Kostakis ID, Papakostidi A, Pistamaltzian N, Ardavanis A (2012). Meta-analysis of the predictive value of KRAS mutations in treatment response using cetuximab in colorectal cancer. J BUON.

[15] Lee GH, Malietzis G, Askari A, Bernardo D, Al-Hassi HO, Clark SK (2015). Is right-sided colon cancer different to left-sided colorectal cancer? - a systematic review. Eur J Surg Oncol.

[16] Moher D, Liberati A, Tetzlaff J, Altman DG, Group P (2009). Preferred reporting items for systematic reviews and meta-analyses: the PRISMA statement. PLoS Med.

[17] Parmar MK, Torri V, Stewart L (1998). Extracting summary statistics to perform meta-analyses of the published literature for survival endpoints. Stat Med.

[18] (2016). http://www.ohri.ca/programs/clinical_epidemiology/oxford.asp.

[19] Higgins Julian PT, Green Sally (2008). Cochrane handbook for systematic reviews of interventions.

[20] Jue W, Ting-ting Z, Juan L, Li-li S, Li B (2016). Comparison of the Prognosis of Different Primary Tumor Location in Patients with K-ras Wild Type Metastatic Colorectal Cancer treated with Cetuximab Combined Chemotherapy. Progress in Modern Biomedicine.

[21] Wang F, Bai L, Liu TS, Yu YY, He MM, Liu KY, Luo HY, Zhang DS, Jin Y, Wang FH, Wang ZQ, Wang DS, Qiu MZ (2015). Right-sided colon cancer and left-sided colorectal cancers respond differently to cetuximab. Chin J Cancer.

[22] von Einem JC, Heinemann V, von Weikersthal LF, Vehling-Kaiser U, Stauch M, Hass HG, Decker T, Klein S, Held S, Jung A, Kirchner T, Haas M, Holch J (2014). Left-sided primary tumors are associated with favorable prognosis in patients with KRAS codon 12/13 wild-type metastatic colorectal cancer treated with cetuximab plus chemotherapy: an analysis of the AIO KRK-0104 trial. J Cancer Res Clin Oncol.

[23] Chen KH, Shao YY, Chen HM, Lin YL, Lin ZZ, Lai MS, Cheng AL, Yeh KH (2016). Primary tumor site is a useful predictor of cetuximab efficacy in the third-line or salvage treatment of KRAS wild-type (exon 2 non-mutant) metastatic colorectal cancer: a nationwide cohort study. BMC Cancer.

[24] Rui Q, Yan S, Li C, Zhiyong W, Yalin H, Guanghai D (2014). Efficacy analysis of cetuximab plus chemotherapy for K-Ras wild-type colorectal cancer with metastases. Chinese Clinical Oncology.

[25] Moretto R, Cremolini C, Rossini D, Pietrantonio F, Battaglin F, Mennitto A, Bergamo F, Loupakis F, Marmorino F, Berenato R, Marsico VA, Caporale M, Antoniotti C (2016). Location of Primary Tumor and Benefit From Anti-Epidermal Growth Factor Receptor Monoclonal Antibodies in Patients With RAS and BRAF Wild-Type Metastatic Colorectal Cancer. Oncologist.

[26] Venook AP, Niedzwiecki D, Innocenti F, Fruth B, Greene C, O’Neil BH, Shaw JE, Atkins JN, Horvath LE, Polite BN, Meyerhardt JA, O’Reilly EM, Goldberg RM (2016). Impact of primary (1{o}) tumor location on overall survival (OS) and progression-free survival (PFS) in patients (pts) with metastatic colorectal cancer (mCRC): Analysis of CALGB/SWOG 80405 (Alliance). ASCO Meeting Abstracts.

[27] Heinemann V, Modest DP, Fischer von Weikersthal L, Decker T, Kiani A, Vehling-Kaiser U, Seipelt G (2014). Gender and tumor location as predictors for efficacy: Influence on endpoints in first-line treatment with FOLFIRI in combination with cetuximab or bevacizumab in the AIO KRK 0306 (FIRE3) trial. ASCO Annual Meeting Proceedings.

[28] Sunakawa Y, Ichikawa W, Tsuji A, Denda T, Segawa Y, Negoro Y, Shimada K, Kochi M, Nakamura M, Kotaka M, Tanioka H, Takagane A, Tani S (2016). Prognostic impact of primary tumor location on survival time in patients (pts) with metastatic colorectal cancer (mCRC) treated with cetuximab plus oxaliplatin-based chemotherapy: A subgroup analysis of the JACCRO CC-05/06. ASCO Meeting Abstracts.

[29] Van Cutsem E, Kohne CH, Folprecht G, Guenther S, Beier F, Papamichael D (2016). Efficacy and safety of first-line cetuximab+ FOLFIRI in older and younger patients (pts) with RAS wild-type (wt) metastatic colorectal cancer (mCRC) in the CRYSTAL study [C]//ASCO Annual Meeting Proceedings. J Clin Oncol (Meeting Abstracts).

[30] Taieb J, Tabernero J, Mini E, Subtil F, Folprecht G, Van Laethem JL, Thaler J, Bridgewater J, Petersen LN, Blons H, Collette L, Van Cutsem E, Rougier P (2014). Oxaliplatin, fluorouracil, and leucovorin with or without cetuximab in patients with resected stage III colon cancer (PETACC-8): an open-label, randomised phase 3 trial. Lancet Oncol.

[31] Tejpar S, Stintzing S, Ciardiello F, Tabernero J, Van Cutsem E, Beier F, Esser R, Lenz HJ, Heinemann V (2016). Prognostic and Predictive Relevance of Primary Tumor Location in Patients With RAS Wild-Type Metastatic Colorectal Cancer: Retrospective Analyses of the CRYSTAL and FIRE-3 Trials. JAMA Oncol.

[32] Nitsche U, Stogbauer F, Spath C, Haller B, Wilhelm D, Friess H, Bader FG (2016). Right Sided Colon Cancer as a Distinct Histopathological Subtype with Reduced Prognosis. Dig Surg.

[33] Qiu LX, Mao C, Zhang J, Zhu XD, Liao RY, Xue K, Li J, Chen Q (2010). Predictive and prognostic value of KRAS mutations in metastatic colorectal cancer patients treated with cetuximab: a meta-analysis of 22 studies. Eur J Cancer.

[34] Berger MD, Stintzing S, Heinemann V, Yang D, Sunakawa Y, Ning Y, Matsusaka S, Okazaki S, Miyamoto Y, Suenaga M, Schirripa M, West JD, Hanna DL (2016). MKNK1 SNP rs8602 to predict outcome for mCRC patients treated with first-line FOLFIRI and bevacizumab: Data from FIRE-3 trial. ASCO Meeting Abstracts.

[35] Hatch AJ, Sibley AB, Starr MD, Brady JC, Jiang C, Jia J, Bowers DL, Pang H, Owzar K, Niedzwiecki D, Innocenti F, Venook AP, Hurwitz HI (2016). Blood-based markers of efficacy and resistance to cetuximab treatment in metastatic colorectal cancer: results from CALGB 80203 (Alliance). Cancer Med.

