# Supplementary material for: The impact of primary tumor location on efficacy of cetuximab in metastatic colorectal cancer patients with different Kras status: a systematic review and meta-analysis
Source: Oncotarget. 2017 Jul 5;8(32):53631–41. doi: 10.18632/oncotarget.19022 (PMC5581136; doi:10.18632/oncotarget.19022)
Supplement: Supplementary file 1 [file oncotarget-08-53631-s001.pdf]

The impact of primary tumor location on efficacy of cetuximab in metastatic colorectal cancer patients with different Kras status: a systematic review and meta-analysis

SUPPLEMENTARY MATERIALS

Supplementary Table 1: Additional baseline characteristics of included studies

Study	Year	stage	Definition of OS/PFS	OS/ PFS	Surgery prior to CT	CR/PR/DCR	RCT or restrospective or observational
Wang Jue	2016	metastatic	Yes	Clear	No	RECIST	Restrospective
Feng Wang	2015	metastatic	Yes	Clear	Partly	RECIST 1.1	Restrospective
von Einem JC	2014	metastatic	Yes	Clear	No	RECIST	Restrospective
Kuo-Hsing Chen	2016	metastatic	Yes	Clear	No	NR	Restrospective
Rui Qin	2014	metastatic	Yes	Clear	No	RECIST 1.1	Restrospective
Moretto R	2016	metastatic	Yes	Clear	No	RECIST	Restrospective
Alan P. Venook	2016	metastatic	Yes	Clear	Partly	RECIST	Restrospective
Eric Van Cutsem	2016	metastatic	Yes	Clear	Partly	RECIST	Restrospective
Heinemann V	2014	metastatic	Yes	Clear	Partly	RECIST 1.1	Restrospective
Yu Sunakawa	2016	metastatic	Yes	Clear	No	RECIST	Restrospective

Note, data in the study of Julien Taieb was removed from meta-analysis as the obvious different stage and treatments.
